# Supplementary material for: Developing small Cas9 hybrids using molecular modeling
Source: Sci Rep. 2024 Jul 26;14:17233. doi: 10.1038/s41598-024-68107-1 (PMC11282279; doi:10.1038/s41598-024-68107-1)
Supplement: Supplementary file 1 — Supplementary Information. [file 41598_2024_68107_MOESM1_ESM.docx]

Supporting information

Developing small Cas9 hybrids using molecular modeling

Antoine Mangin^1^, Vincent Dion^1*^ , and Georgina Menzies^2*^

1: UK Dementia Research Institute at Cardiff University, CF24 4HQ, UK

2: School of Biosciences, Cardiff University, Cardiff CF10 3AX, UK

*Corresponding authors: [dionv@cardiff.ac.uk](mailto:dionv@cardiff.ac.uk), [menziesg@cardiff.ac.uk](mailto:menziesg@cardiff.ac.uk)


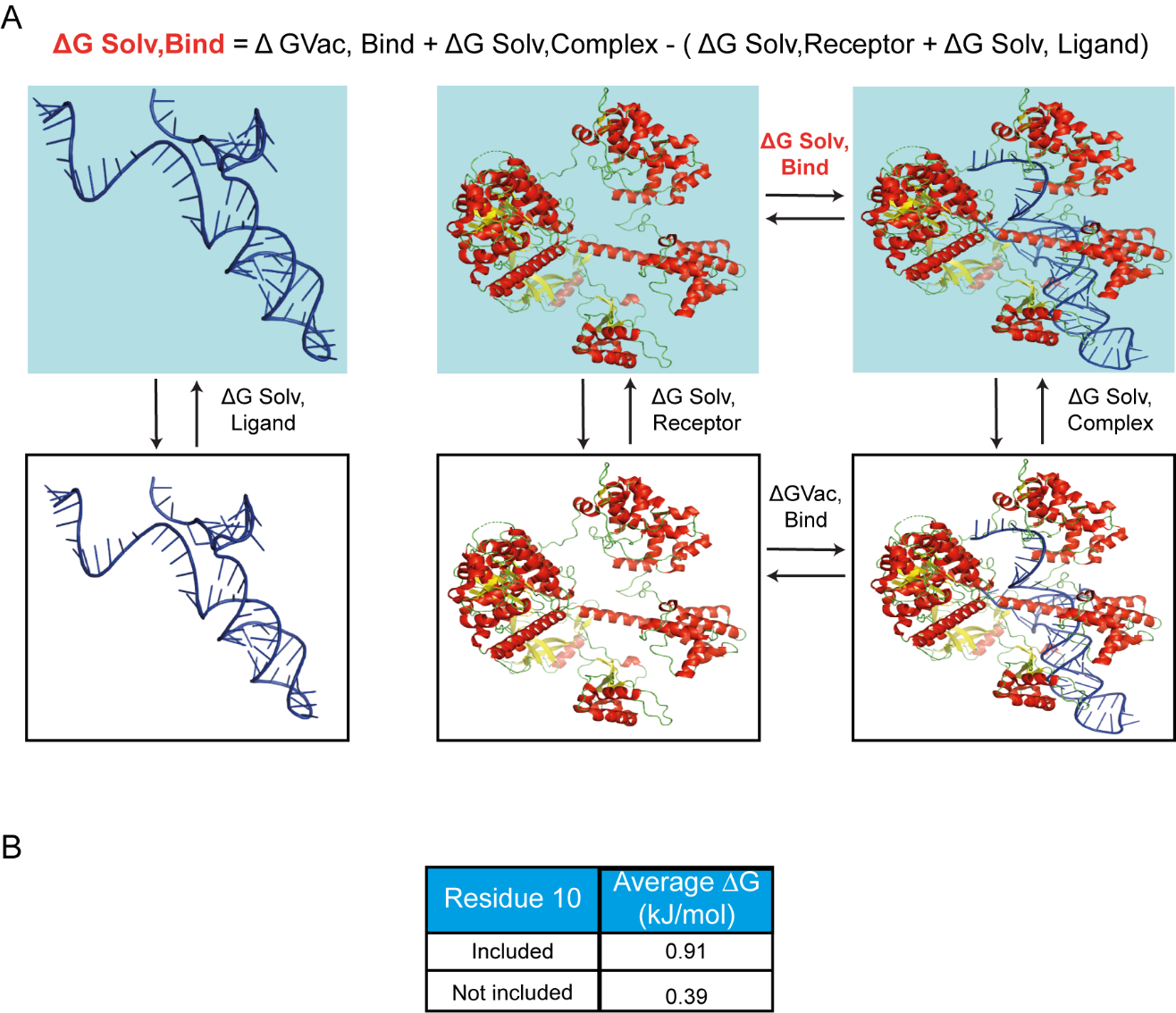


**Supplementary figure 1:** **Binding energy calculations between the Cas9 protein and its sgRNA.** A) Principle behind binding energy calculations. The blue boxes represent the system in water whereas the white boxes are in vacuum. B) An average of the ΔG binding for each residue between the nickase and nuclease. ‘Included’ means that the average ΔG was calculated for every residue in the Cas9 protein, whereas ‘not included’ excludes the amino acid that changes between the nuclease (D10) and the nickase (A10). This shows an outsize contribution of this residue to the overall binding energy of the protein.


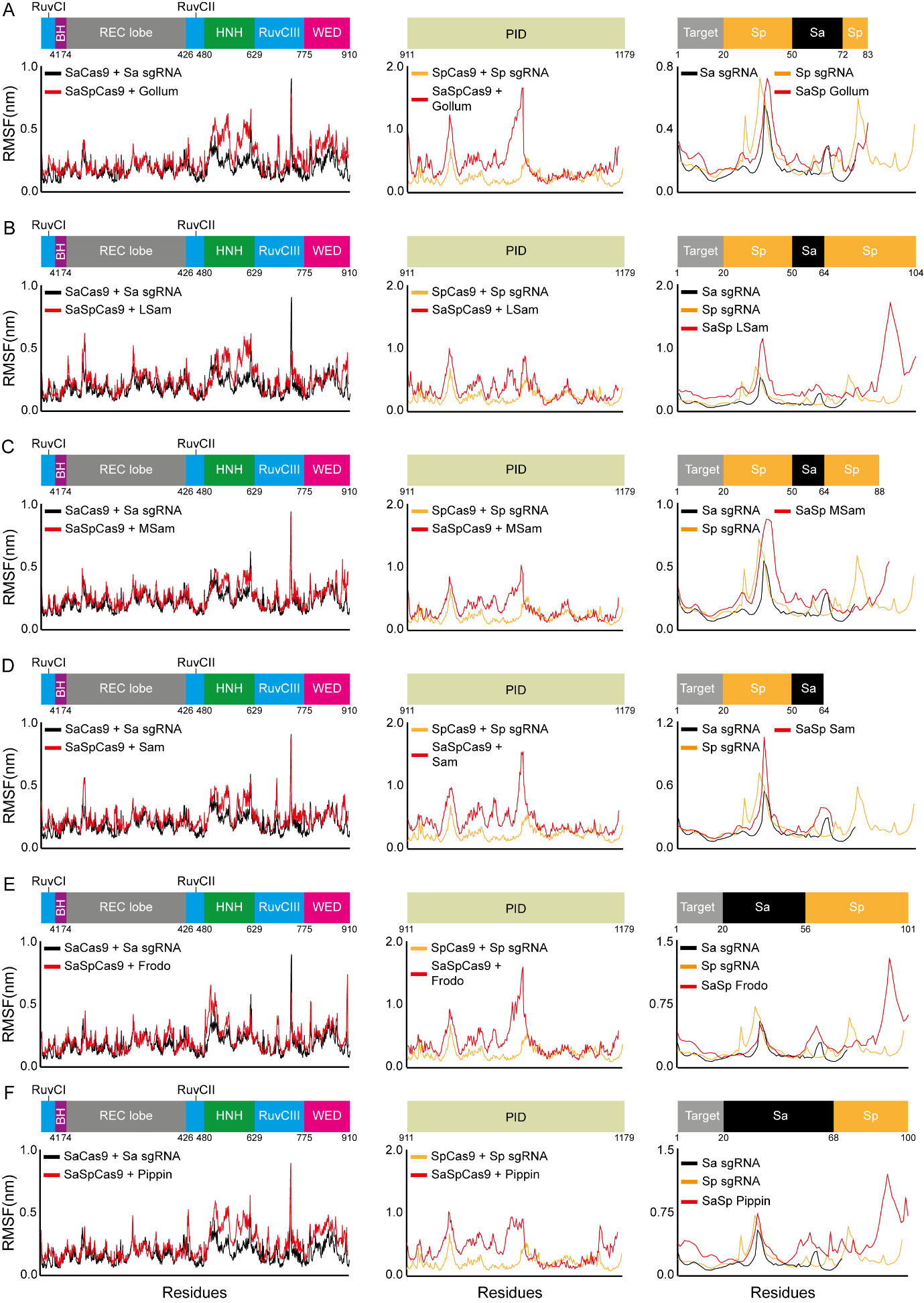


**Supplementary figure 2: RMSF analysis from molecular dynamics simulations for the SaSp hybrids**: A - F) RMSF of SaSp hybrids (red)**,** SaCas9 (black), and SpCas9 (orange) together with their cognate sgRNAs. A) Gollum, B) LSam, C) MSam, D) Sam, E) Frodo, F) Pippin. Left: N-terminal, middle: PID, right sgRNA. This figure shows that all the hybrid pairs are expected to be stable.


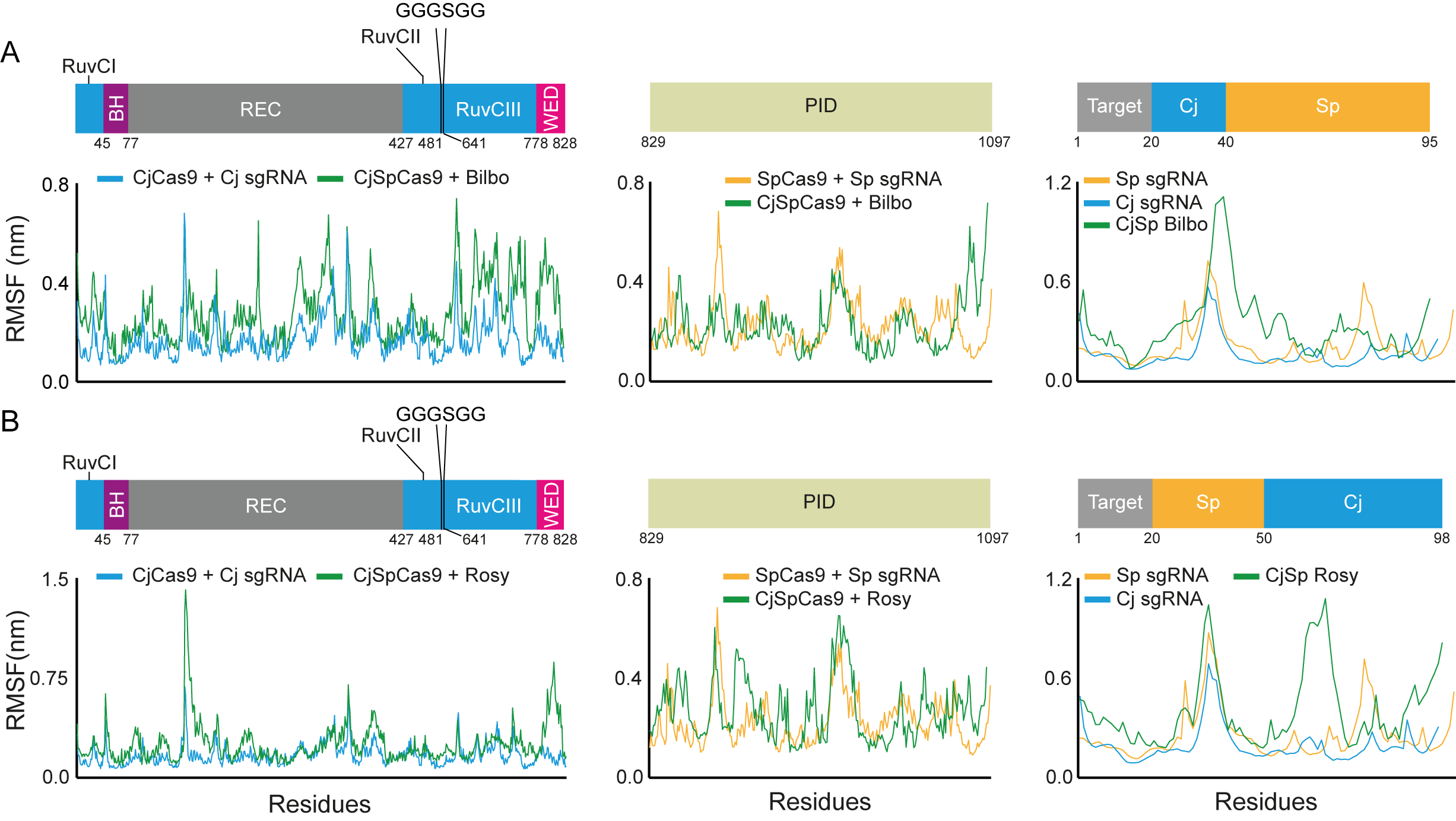


**Supplementary figure 3:** **RMSF analysis from molecular dynamics simulations for the CjSp hybrids**: A - B) RMSF of CjSp hybrids (green) **,** CjCas9 (blue) and SpCas9 (orange), together with their cognate sgRNAs. A) Bilbo, B) Rosy. Left: N-terminal, middle: PID, right sgRNA. This figure shows that all the hybrid pairs are expected to be stable.


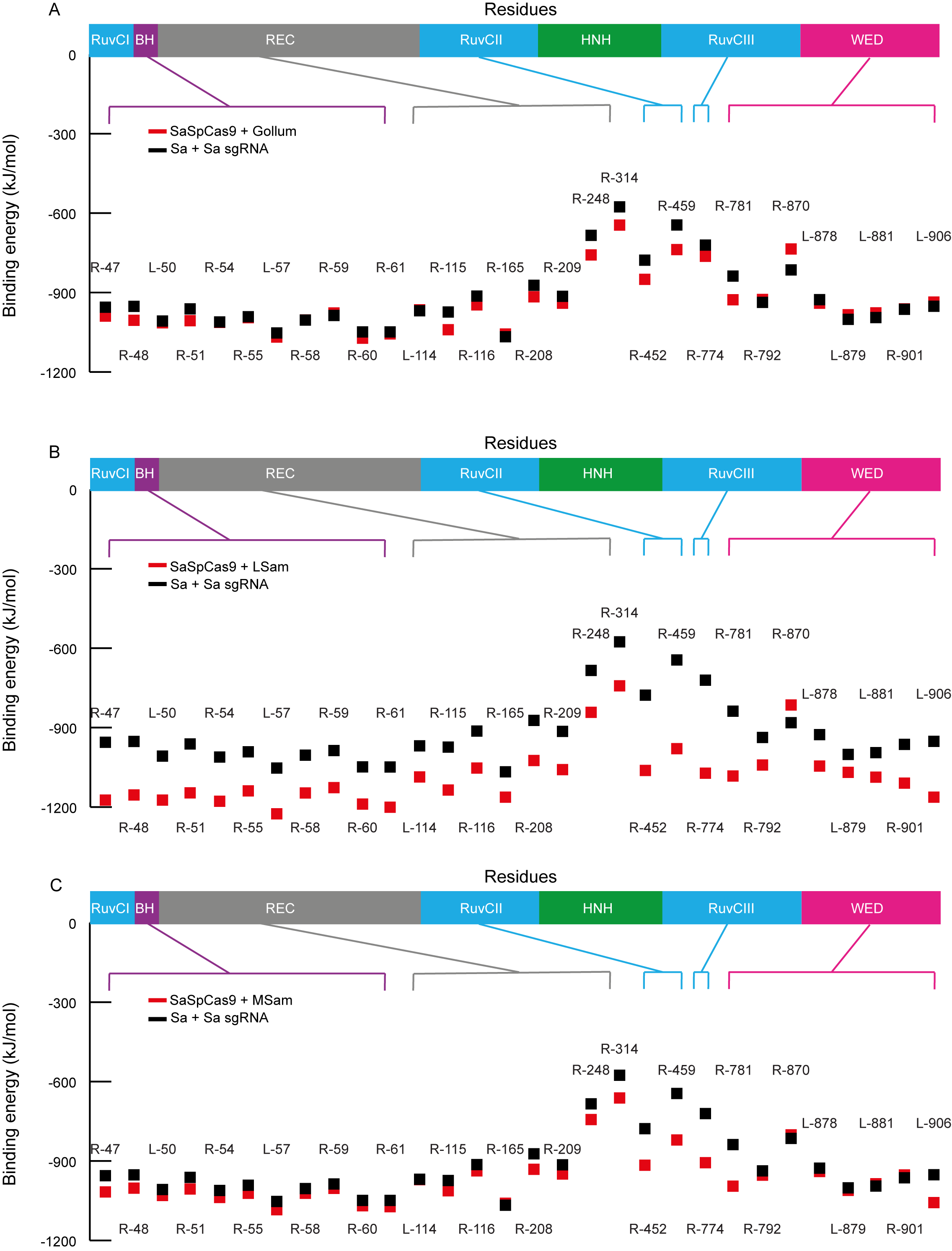


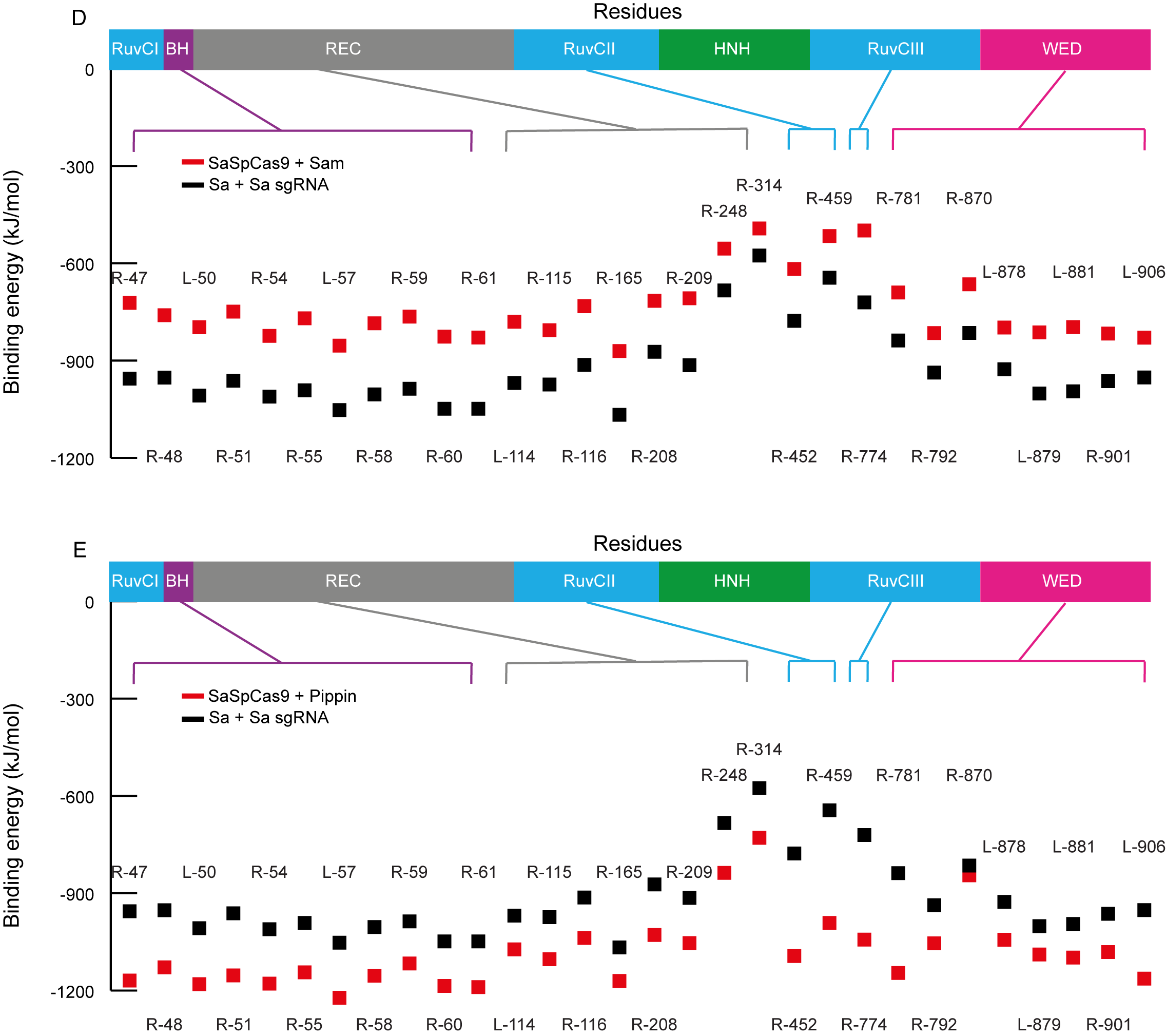


**Supplementary figure 4: Binding energy calculation for the SaSpCas9 hybrids.** The binding energy for each amino acid known to interact with the sgRNA from the crystal structures are shown for comparing A) SaCas9 (black) to SaSpCas9 (red). A) Gollum, B) LSam, C) MSam, D) Pippin, E) Sam. This figures shows that the sgRNAs are expected to make the contacts with the sgRNAs as predicted from the structural information available.


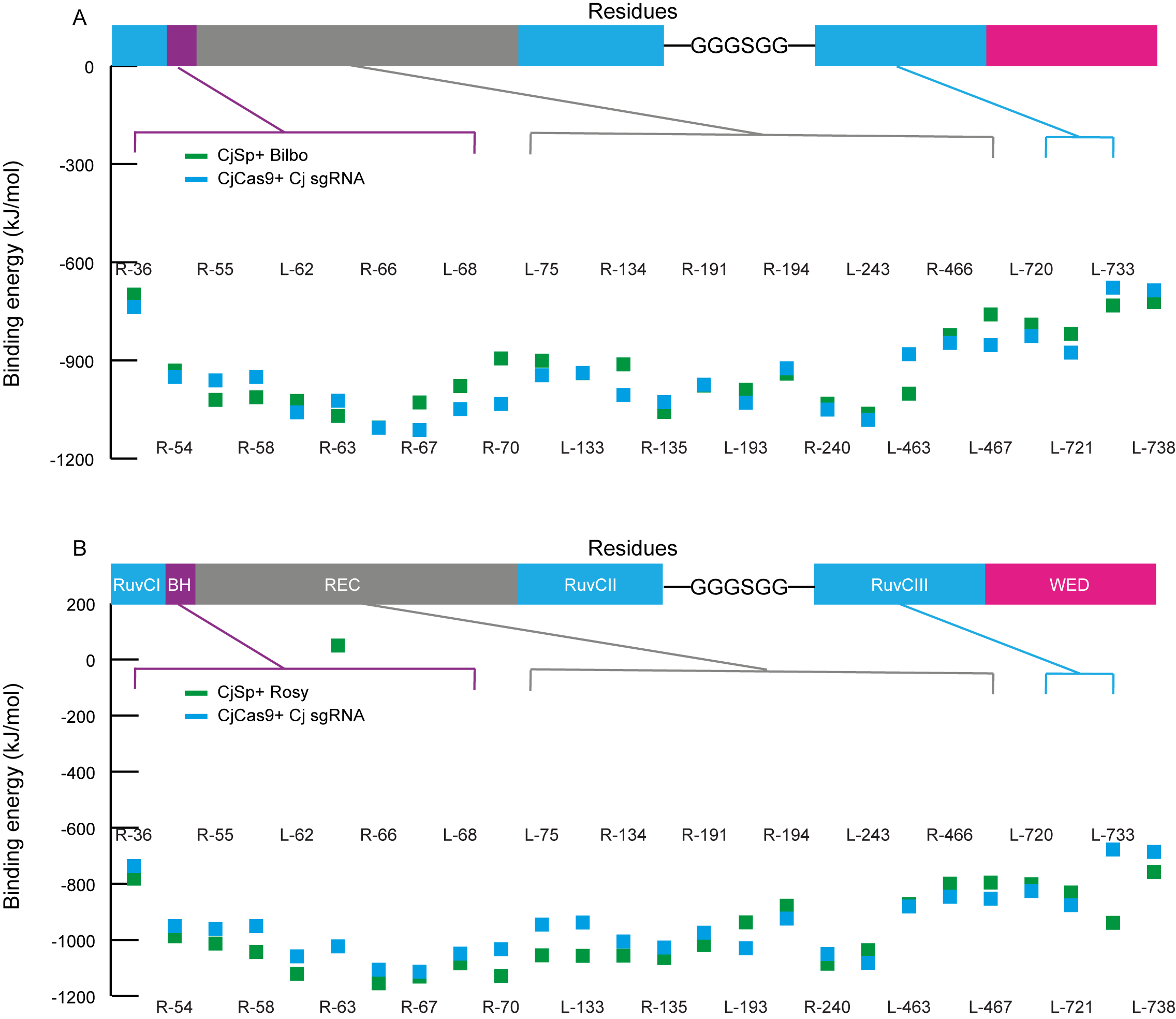


**Supplementary figure 5:** **Binding energy calculation for the CjSPCas9 hybrids.** The binding energy for each amino acid known to interact with the sgRNA from the crystal structures are shown for comparing CjCas9 (blue) to CjSpCas9 (green). A) Bilbo, B) Rosy. This figures shows that the sgRNAs are expected to make the contacts with the sgRNAs as predicted from the structural information available.


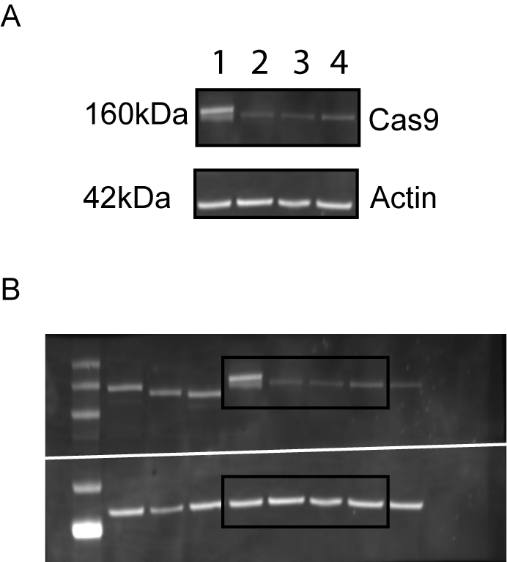


**Supplementary figure 6: Expression of Cas9 hybrids in HEK cells:** A) Expression of 1) SpCas9 together with its sgRNA, 2) SpCas9 with Gollum, 3) SaSpCas9 plus Frodo, and 4) SaSpCas9 alongside Merry. B) Uncropped western blot for Cas9 expression. Black boxes indicate where the blots were cropped. Note that the membrane was cut prior to blotting but imaged together, which is why the top and bottom parts of the gel look different. The white line was added on top of the image to highlight this as per the journal’s policy.


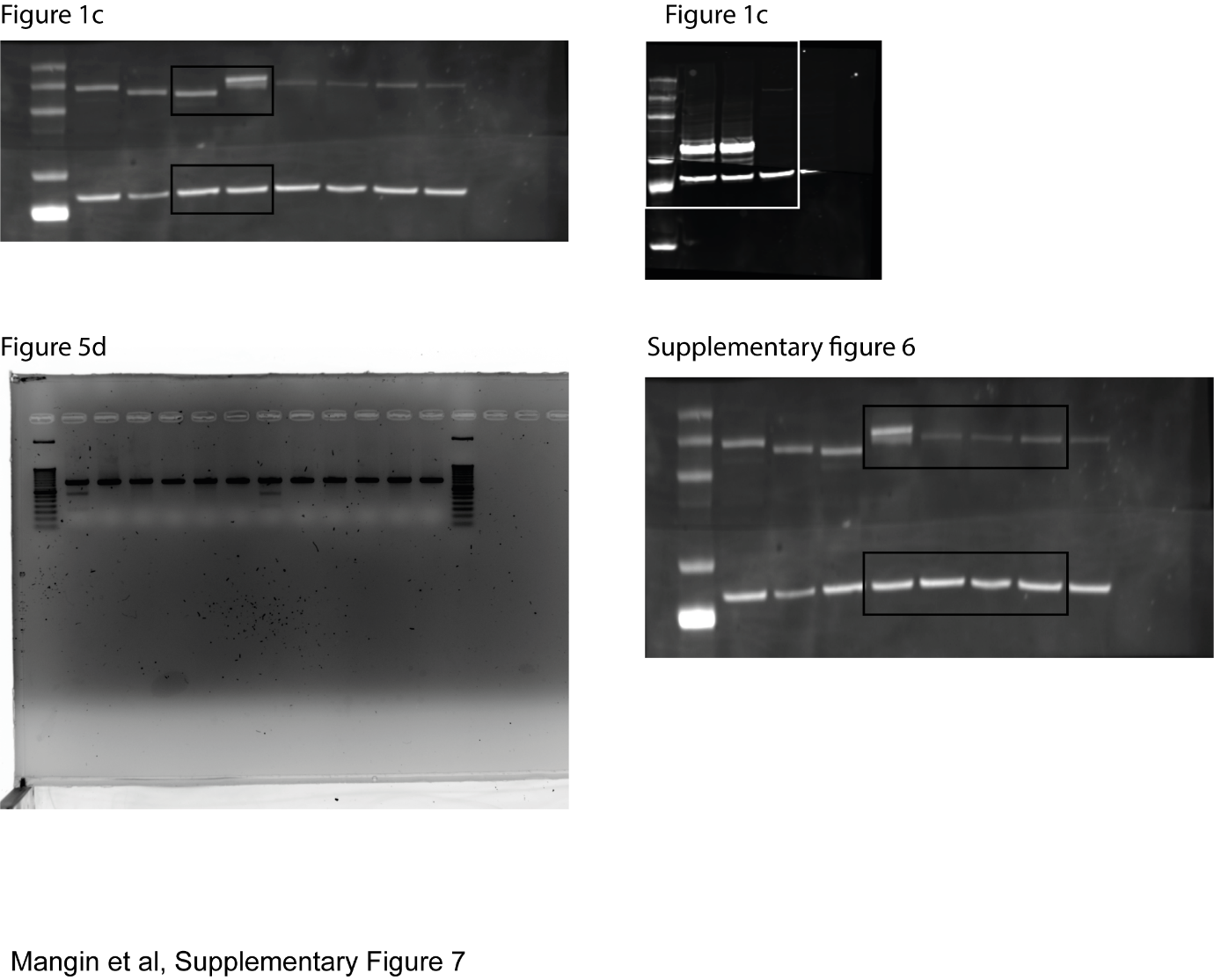


**Supplementary figure 7: Unaltered blots and gels**. Uncropped western blots and gels for all those presented in this study.

**Supplementary Table S1:** Amino acid sequence of Cas9 orthologues and of the Cas9 hybrids.

| Cas9 | Protein sequence (PID in RED, Nickase mutation in BLUE) | Size (AA) |
| --- | --- | --- |
| Sp | MDKKYSIGL**A**IGTNSVGWAVITDEYKVPSKKFKVLGNTDRHSIKKNLIGALLFDSGETAE  ATRLKRTARRRYTRRKNRICYLQEIFSNEMAKVDDSFFHRLEESFLVEEDKKHERHPIFG  NIVDEVAYHEKYPTIYHLRKKLVDSTDKADLRLIYLALAHMIKFRGHFLIEGDLNPDNSD  VDKLFIQLVQTYNQLFEENPINASGVDAKAILSARLSKSRRLENLIAQLPGEKKNGLFGN  LIALSLGLTPNFKSNFDLAEDAKLQLSKDTYDDDLDNLLAQIGDQYADLFLAAKNLSDAI  LLSDILRVNTEITKAPLSASMIKRYDEHHQDLTLLKALVRQQLPEKYKEIFFDQSKNGYA  GYIDGGASQEEFYKFIKPILEKMDGTEELLVKLNREDLLRKQRTFDNGSIPHQIHLGELH  AILRRQEDFYPFLKDNREKIEKILTFRIPYYVGPLARGNSRFAWMTRKSEETITPWNFEE  VVDKGASAQSFIERMTNFDKNLPNEKVLPKHSLLYEYFTVYNELTKVKYVTEGMRKPAFL  SGEQKKAIVDLLFKTNRKVTVKQLKEDYFKKIECFDSVEISGVEDRFNASLGTYHDLLKI  IKDKDFLDNEENEDILEDIVLTLTLFEDREMIEERLKTYAHLFDDKVMKQLKRRRYTGWG  RLSRKLINGIRDKQSGKTILDFLKSDGFANRNFMQLIHDDSLTFKEDIQKAQVSGQGDSL  HEHIANLAGSPAIKKGILQTVKVVDELVKVMGRHKPENIVIEMARENQTTQKGQKNSRER  MKRIEEGIKELGSQILKEHPVENTQLQNEKLYLYYLQNGRDMYVDQELDINRLSDYDVDH  IVPQSFLKDDSIDNKVLTRSDKNRGKSDNVPSEEVVKKMKNYWRQLLNAKLITQRKFDNL  TKAERGGLSELDKAGFIKRQLVETRQITKHVAQILDSRMNTKYDENDKLIREVKVITLKS  KLVSDFRKDFQFYKVREINNYHHAHDAYLNAVVGTALIKKYPKLESEFVYGDYKVYDVRK  MIAKSEQEIGKATAKYFFYSNIMNFFKTEITLANGEIRKRPLIETNGETGEIVWDKGRDF  ATVRKVLSMPQVNIVKKTEVQTGGFSKESILPKRNSDKLIARKKDWDPKKYGGFDSPTVA  YSVLVVAKVEKGKSKKLKSVKELLGITIMERSSFEKNPIDFLEAKGYKEVKKDLIIKLPK  YSLFELENGRKRMLASAGELQKGNELALPSKYVNFLYLASHYEKLKGSPEDNEQKQLFVE  QHKHYLDEIIEQISEFSKRVILADANLDKVLSAYNKHRDKPIREQAENIIHLFTLTNLGA  PAAFKYFDTTIDRKRYTSTKEVLDATLIHQSITGLYETRIDLSQLGGD | 1368 |
| Sa | MKRNYILGL**A**IGITSVGYGIIDYETRDVIDAGVRLFKEANVENNEGRRSKRGARRLKRRR  RHRIQRVKKLLFDYNLLTDHSELSGINPYEARVKGLSQKLSEEEFSAALLHLAKRRGVHN  VNEVEEDTGNELSTKEQISRNSKALEEKYVAELQLERLKKDGEVRGSINRFKTSDYVKEA  KQLLKVQKAYHQLDQSFIDTYIDLLETRRTYYEGPGEGSPFGWKDIKEWYEMLMGHCTYF  PEELRSVKYAYNADLYNALNDLNNLVITRDENEKLEYYEKFQIIENVFKQKKKPTLKQIA  KEILVNEEDIKGYRVTSTGKPEFTNLKVYHDIKDITARKEIIENAELLDQIAKILTIYQS  SEDIQEELTNLNSELTQEEIEQISNLKGYTGTHNLSLKAINLILDELWHTNDNQIAIFNR  LKLVPKKVDLSQQKEIPTTLVDDFILSPVVKRSFIQSIKVINAIIKKYGLPNDIIIELAR  EKNSKDAQKMINEMQKRNRQTNERIEEIIRTTGKENAKYLIEKIKLHDMQEGKCLYSLEA  IPLEDLLNNPFNYEVDHIIPRSVSFDNSFNNKVLVKQEENSKKGNRTPFQYLSSSDSKIS  YETFKKHILNLAKGKGRISKTKKEYLLEERDINRFSVQKDFINRNLVDTRYATRGLMNLL  RSYFRVNNLDVKVKSINGGFTSFLRRKWKFKKERNKGYKHHAEDALIIANADFIFKEWKK  LDKAKKVMENQMFEEKQAESMPEIETEQEYKEIFITPHQIKHIKDFKDYKYSHRVDKKPN  RELINDTLYSTRKDDKGNTLIVNNLNGLYDKDNDKLKKLINKSPEKLLMYHHDPQTYQKL  KLIMEQYGDEKNPLYKYYEETGNYLTKYSKKDNGPVIKKIKYYGNKLNAHLDITDDYPNS  RNKVVKLSLKPYRFDVYLDNGVYKFVTVKNLDVIKKENYYEVNSKCYEEAKKLKKISNQA  EFIASFYNNDLIKINGELYRVIGVNNDLLNRIEVNMIDITYREYLENMNDKRPPRIIKTI  ASKTQSIKKYSTDILGNLYEVKSKKHPQIIKKG | 1053 |
| CJ | MARILAF**A**IGISSIGWAFSENDELKDCGVRIFTKVENPKTGESLALPRRLARSARKRLAR  RKARLNHLKHLIANEFKLNYEDYQSFDESLAKAYKGSLISPYELRFRALNELLSKQDFAR  VILHIAKRRGYDDIKNSDDKEKGAILKAIKQNEEKLANYQSVGEYLYKEYFQKFKENSKE  FTNVRNKKESYERCIAQSFLKDELKLIFKKQREFGFSFSKKFEEEVLSVAFYKRALKDFS  HLVGNCSFFTDEKRAPKNSPLAFMFVALTRIINLLNNLKNTEGILYTKDDLNALLNEVLK  NGTLTYKQTKKLLGLSDDYEFKGEKGTYFIEFKKYKEFIKALGEHNLSQDDLNEIAKDIT  LIKDEIKLKKALAKYDLNQNQIDSLSKLEFKDHLNISFKALKLVTPLMLEGKKYDEACNE  LNLKVAINEDKKDFLPAFNETYYKDEVTNPVVLRAIKEYRKVLNALLKKYGKVHKINIEL  AREVGKNHSQRAKIEKEQNENYKAKKDAELECEKLGLKINSKNILKLRLFKEQKEFCAYS  GEKIKISDLQDEKMLEIDHIYPYSRSFDDSYMNKVLVFTKQNQEKLNQTPFEAFGNDSAK  WQKIEVLAKNLPTKKQKRILDKNYKDKEQKNFKDRNLNDTRYIARLVLNYTKDYLDFLPL  SDDENTKLNDTQKGSKVHVEAKSGMLTSALRHTWGFSAKDRNNHLHHAIDAVIIAYANNS  IVKAFSDFKKEQESNSAELYAKKISELDYKNKRKFFEPFSGFRQKVLDKIDEIFVSKPER  KKPSGALHEETFRKEEEFYQSYGGKEGVLKALELGKIRKVNGKIVKNGDMFRVDIFKHKK  TNKFYAVPIYTMDFALKVLPNKAVARSKKGEIKDWILMDENYEFCFSLYKDSLILIQTKD  MQEPEFVYYNAFTSSTVSLIVSKHDNKFETLSKNQKILFKNANEKEVIAKSIGIQNLKVF  EKYIVSALGEVTKAEFRQREDFKK | 984 |
| SaSp | MKRNYILGL**A**IGITSVGYGIIDYETRDVIDAGVRLFKEANVENNEGRRSKRGARRLKRRR  RHRIQRVKKLLFDYNLLTDHSELSGINPYEARVKGLSQKLSEEEFSAALLHLAKRRGVHN  VNEVEEDTGNELSTKEQISRNSKALEEKYVAELQLERLKKDGEVRGSINRFKTSDYVKEA  KQLLKVQKAYHQLDQSFIDTYIDLLETRRTYYEGPGEGSPFGWKDIKEWYEMLMGHCTYF  PEELRSVKYAYNADLYNALNDLNNLVITRDENEKLEYYEKFQIIENVFKQKKKPTLKQIA  KEILVNEEDIKGYRVTSTGKPEFTNLKVYHDIKDITARKEIIENAELLDQIAKILTIYQS  SEDIQEELTNLNSELTQEEIEQISNLKGYTGTHNLSLKAINLILDELWHTNDNQIAIFNR  LKLVPKKVDLSQQKEIPTTLVDDFILSPVVKRSFIQSIKVINAIIKKYGLPNDIIIELAR  EKNSKDAQKMINEMQKRNRQTNERIEEIIRTTGKENAKYLIEKIKLHDMQEGKCLYSLEA  IPLEDLLNNPFNYEVDHIIPRSVSFDNSFNNKVLVKQEENSKKGNRTPFQYLSSSDSKIS  YETFKKHILNLAKGKGRISKTKKEYLLEERDINRFSVQKDFINRNLVDTRYATRGLMNLL  RSYFRVNNLDVKVKSINGGFTSFLRRKWKFKKERNKGYKHHAEDALIIANADFIFKEWKK  LDKAKKVMENQMFEEKQAESMPEIETEQEYKEIFITPHQIKHIKDFKDYKYSHRVDKKPN  RELINDTLYSTRKDDKGNTLIVNNLNGLYDKDNDKLKKLINKSPEKLLMYHHDPQTYQKL  KLIMEQYGDEKNPLYKYYEETGNYLTKYSKKDNGPVIKKIKYYGNKLNAHLDITDDYPNS  RNKVVKLSTGGFSKESILPKRNSDKLIARKKDWDPKKYGGFDSPTVAYSVLVVAKVEKGK  SKKLKSVKELLGITIMERSSFEKNPIDFLEAKGYKEVKKDLIIKLPKYSLFELENGRKRM  LASAGELQKGNELALPSKYVNFLYLASHYEKLKGSPEDNEQKQLFVEQHKHYLDEIIEQI  SEFSKRVILADANLDKVLSAYNKHRDKPIREQAENIIHLFTLTNLGAPAAFKYFDTTIDR  KRYTSTKEVLDATLIHQSITGLYETRIDLSQLGGD | 1179 |
| CjSp | MARILAF**A**IGISSIGWAFSENDELKDCGVRIFTKVENPKTGESLALPRRLARSARKRLAR  RKARLNHLKHLIANEFKLNYEDYQSFDESLAKAYKGSLISPYELRFRALNELLSKQDFAR  VILHIAKRRGYDDIKNSDDKEKGAILKAIKQNEEKLANYQSVGEYLYKEYFQKFKENSKE  FTNVRNKKESYERCIAQSFLKDELKLIFKKQREFGFSFSKKFEEEVLSVAFYKRALKDFS  HLVGNCSFFTDEKRAPKNSPLAFMFVALTRIINLLNNLKNTEGILYTKDDLNALLNEVLK  NGTLTYKQTKKLLGLSDDYEFKGEKGTYFIEFKKYKEFIKALGEHNLSQDDLNEIAKDIT  LIKDEIKLKKALAKYDLNQNQIDSLSKLEFKDHLNISFKALKLVTPLMLEGKKYDEACNE  LNLKVAINEDKKDFLPAFNETYYKDEVTNPVVLRAIKEYRKVLNALLKKYGKVHKINIEL  AREVGKNHSQRAKIEKEQNENYKAKKDAELECEKLGLKINSKNILKLRLFKEQKEFCAYS  GEKIKISDLQDEKMLEIDHIYPYSRSFDDSYMNKVLVFTKQNQEKLNQTPFEAFGNDSAK  WQKIEVLAKNLPTKKQKRILDKNYKDKEQKNFKDRNLNDTRYIARLVLNYTKDYLDFLPL  SDDENTKLNDTQKGSKVHVEAKSGMLTSALRHTWGFSAKDRNNHLHHAIDAVIIAYANNS  IVKAFSDFKKEQESNSAELYAKKISELDYKNKRKFFEPFSGFRQKVLDKIDEIFVSKPER  KKPSGALHEETFRKEEEFYQSYGGKEGVLKALELGKIRKVNGKIVKNGTGGFSKESILPK  RNSDKLIARKKDWDPKKYGGFDSPTVAYSVLVVAKVEKGKSKKLKSVKELLGITIMERSS  FEKNPIDFLEAKGYKEVKKDLIIKLPKYSLFELENGRKRMLASAGELQKGNELALPSKYV  NFLYLASHYEKLKGSPEDNEQKQLFVEQHKHYLDEIIEQISEFSKRVILADANLDKVLSA  YNKHRDKPIREQAENIIHLFTLTNLGAPAAFKYFDTTIDRKRYTSTKEVLDATLIHQSIT  GLYETRIDLSQLGGD | 1097 |

**Supplementary Table 2:** Sequence of the WT sgRNAs and hybrid sgRNAs.

| sgRNA | DNA sequence (Yellow = Sp sgRNA ; Black = Sa sgRNA ; Blue = Cj sgRNA; Grey= Target sequence) | Size (bp) |
| --- | --- | --- |
| Sp sgRNA | GCUGCUGCUGCUGCUGCUGGUUUUAGAGCUAGAAAAUAGCAAGUUAAAAUAAGGCUAGUCCGUUAUCAACUUGAAAAAGUGGCACCGAGUCGGUGCUU | 98 |
| Sa sgRNA | GCUGCUGCUGCUGCUGCUGGUUUUAGUACUCUGGAAACAGAAUCUACUAAAACAAGGCAAAAUGCCGUGUUUAUCUCGUCAACUUGUUGGCGAGAU | 96 |
| Cj sgRNA | GCUGCUGCUGCUGCUGCUGGUUUUAGUCCCUGAAAAAGGGACUAAAAUAAAGAGUUUGCGGGACUCUGCGGGGUUACAAUCCCCUAAAACCGC | 93 |
| Frodo sgRNA | GCUGCUGCUGCUGCUGCUGGUUUUAGUACUCUGGAAACAGAAUCUACUAAAACAAGGCUAGUCCGUUAUCAACUUGAAAAAGUGGCACCGAGUCGGUGCU | 100 |
| Sam sgRNA | GCUGCUGCUGCUGCUGCUGGUUUUAGAGCUAGAAAUAGCAAGUUAAAAUAAGGCAAAAUGCCG | 63 |
| Medium Sam sgRNA | GCUGCUGCUGCUGCUGCUGGUUUUAGAGCUAGAAAUAGCAAGUUAAAAUAAGGCAAAAUGCCGAUCUCGUCAACUUGUUGGCGAGAU | 87 |
| Large Sam sgRNA | GCUGCUGCUGCUGCUGCUGGUUUUAGAGCUAGAAAUAGCAAGUUAAAAUAAGGCAAAAUGCCGAUCUCGUCAACUUGUUGCGAGAUGCACCGAGUCGGUGCUU | 103 |
| Pippin sgRNA | GCUGCUGCUGCUGCUGCUGGUUUUAGUACUCUGGAAACAGAAUCUACUAAAACAAGGCAAAAUGCCGAACUUGAAAAAGUGGCCACCGAGUCGGUGCUU | 99 |
| Merry sgRNA | GCUGCUGCUGCUGCUGCUGGUUUUAGUACUCUGGAAACAGAAUCUACUAAAACAAGGCAAAAUGCCGAACUUGAAAAAGUG | 81 |
| Gollum sgRNA | GCUGCUGCUGCUGCUGCUGCGUUUUAGAGCUAGAAAUAGCAAGUUAAAAUAAGGCAAAAUGCCGUGUUUAACUUGAAAAAGUG | 83 |
| Bilbo sgRNA | GCUGCUGCUGCUGCUGCUGGUUUUAGUCCCUGAAAAGGGACUAAAAUGGCUAGUCCGUUAUCAACUUGAAAAAGUGGCACCGAGUCGGUGCU | 92 |
| Rosy sgRNA | GCUGCUGCUGCUGCUGCUGGUUUUAGAGCUAGAAAUAGCAAGUUAAAAUAAAGAGUUUGCGGGACUCUGCGGGGUUACAAUCCCCUAAAACCGC | 94 |

**Supplementary Table 3:** List of plasmids used.

| Plasmid name | Content | Reference | Addgene ID number |
| --- | --- | --- | --- |
| pcDNA3.3-TOPO - Cas9_D10A | Sp Cas9 D10A | ^1^ | 41816 |
| pPN10-gCTG | Sp Cas9 sgRNA sgCTG | ^2^ | 114385 |
| pUC57-ITR2-H1- sgCTG Frodo-eCMV-SaSpD10A (Neuron codon optimization)-3XFLAG-SV40 NLS-ITR2 | SaSpD10A Cas9+ Frodo sgCTG | This study | 210734 |
| pUC57-ITR2-H1- sgCTG Gollum-eCMV-SaSpD10A (Neuron codon optimization)-3XFLAG-SV40 NLS-ITR2 | SaSpD10A Cas9+ Gollum sgCTG | This study | 210735 |
| pUC57-ITR2-H1- sgCTG Merry-eCMV-SaSpD10A+linker (Neuron codon optimization)-3XFLAG-SV40 NLS-ITR2 | SaSpD10A Cas9 + Merry sgCTG | This study | 210736 |
| pUC57-ITR2-H1-GFP sgRNA Target 1- eCMV- S.pyoegenes-3XFLAG-SV40NLS-ITR2 | Sp Cas9 + Sp guide targeting GFP1 | This study | 210737 |
| pUC57-ITR2-H1-GFP sgRNA Target 2- eCMV- S.pyoegenes-3XFLAG-SV40NLS-ITR2 | Sp Cas9 + Sp guide targeting EGFP2 | This study | 210738 |
| pUC57-ITR2-H1-GFP sgRNA Target 3- eCMV- S.pyoegenes-3XFLAG-SV40NLS-ITR2 | Sp Cas9 + Sp guide targeting EGFP3 | This study | 210739 |
| pUC57-ITR2-H1-GFP sgRNA Target 4- eCMV- S.pyoegenes-3XFLAG-SV40NLS-ITR2 | Sp Cas9 + Sp guide targeting EGFP4 | This study | 210740 |
| pUC57-ITR2-H1-GFP sgRNA Merry Target 1- eCMV- SaSp-3XFLAG-SV40NLS-ITR2 | SaSp Cas9 + Merry guide targeting EGFP1 | This study | 210741 |
| pUC57-ITR2-H1-GFP sgRNA Merry Target 2- eCMV- SaSp-3XFLAG-SV40NLS-ITR2 | SaSp Cas9 + Merry guide targeting EGFP2 | This study | 210742 |
| pUC57-ITR2-H1-GFP sgRNA Merry Target 3- eCMV- SaSp-3XFLAG-SV40NLS-ITR2 | SaSp Cas9 + Merry guide targeting EGFP3 | This study | 210743 |
| pUC57-ITR2-H1-GFP sgRNA Merry Target 4- eCMV- SaSp-3XFLAG-SV40NLS-ITR2 | SaSp Cas9 + Merry guide targeting EGFP4 | This study | 210744 |
| pUC57-ITR2-H1-GFP sgRNA Gollum Target 1- eCMV- SaSp-3XFLAG-SV40NLS-ITR2 | SaSp Cas9 + Gollum guide targeting EGFP1 | This study | 210745 |
| pUC57-ITR2-H1-GFP sgRNA Gollum Target 2- eCMV- SaSp-3XFLAG-SV40NLS-ITR2 | SaSp Cas9 + Gollum guide targeting EGFP2 | This study | 210746 |
| pUC57-ITR2-H1-GFP sgRNA Gollum Target 3- eCMV- SaSp-3XFLAG-SV40NLS-ITR2 | SaSp Cas9 + Gollum guide targeting EGFP3 | This study | 210747 |
| pUC57-ITR2-H1-GFP sgRNA Gollum Target 4- eCMV- SaSp-3XFLAG-SV40NLS-ITR2 | SaSp Cas9 + Gollum guide targeting EGFP4 | This study | 210748 |
| pUC57-ITR2-H1- sgCTG Bilbo -eCMV-CjSpD8A-3XFLAG-SV40 NLS-ITR2 | CjSpD8A Cas9 + Bilbo sgCTG | This study | 210749 |
| pUC57-ITR2-H1-sgCTG-SlugD10A-3XFLAG-SV40 NLS-ITR2 | SlugD10A Cas9 + sgCTG | This study | 222857 |
| pUC57-ITR2-H1-sgCAG-SlugD10A-3XFLAG-SV40 NLS-ITR2 | SlugD10A Cas9 + sgCAG | This study | 222858 |
| pUC57-ITR2-H1-sgAGC-SlugD10A-3XFLAG-SV40 NLS-ITR2 | SlugD10A Cas9 + sgAGC | This study | 222859 |
| pUC57-ITR2-H1-sgGCA-SlugD10A-3XFLAG-SV40 NLS-ITR2 | SlugD10A Cas9 + sgGCA | This study | 222860 |
| pcDNA3.1-CMV-SV40NLS-OgeuIscBE193A-3XHA | OgeuIscBE193A | This study | 222861 |
| OgeuIscB GCT RNA | ω-RNA GCT | This study | 222862 |
| OgeuIscB TGC RNA | ω-RNA TGC | This study | 222863 |
| OgeuIscB GCA RNA | ω-RNA GCA | This study | 222864 |
| OgeuIscB CTG RNA | ω-RNA CTG | This study | 222865 |
| OgeuIscB AGC RNA | ω-RNA AGC | This study | 222866 |
| OgeuIscB CAG RNA | ω-RNA CAG | This study | 222867 |

**Supplementary Table S4:** The sgRNA sequence used to target GFP.

| Target | Sequence | Reference |
| --- | --- | --- |
| GFP Target 1 | GGGCACGGGCAGCUUGCCGG | ^3^ |
| GFP Target 2 | GAUGCCGUUCUUCUGCUUGU | ^3^ |
| GFP Target 3 | GGUGGUGCAGAUGAACUUCA | ^3^ |
| GFP Target 4 | GGGCGAGGAGCUGUUCACCG | ^3^ |

**Supplementary Table S5:** The primers used to amplify the region targeted by the GFP sgRNA

| Name | Description | Sequence |
| --- | --- | --- |
| oVIN 3474 | Forward primer to amplify the target 1,3 and 4 for T7 endonuclease I assay | ACGCTGTTTTGACCTCCAT |
| oVIN 3475 | Reverse primer to amplify the target 1,3 and 4 for T7 endonuclease I assay | CGTCGTCCTTGAAGAAGATG |
| oVIN 3476 | Reverse primer to amplify the target 2 for T7 endonuclease I assay | GTCTTTGCTCAGGGCGGACT |
| oVIN 3477 | Forward primer to amplify the target 2 for T7 endonuclease I assay | TTTACCCTAGCACGCATGAAG |

**References:**

1. Mali, P. *et al.* RNA-Guided Human Genome Engineering via Cas9. *Science* **339**, 823–826 (2013).

2. Cinesi, C., Aeschbach, L., Yang, B. & Dion, V. Contracting CAG/CTG repeats using the CRISPR-Cas9 nickase. *Nat Commun* **7**, 13272 (2016).

3. Fu, Y., Sander, J. D., Reyon, D., Cascio, V. M. & Joung, J. K. Improving CRISPR-Cas nuclease specificity using truncated guide RNAs. *Nat Biotechnol* **32**, 279–284 (2014).
